# Therapeutic potential of GSK-J4, a histone demethylase KDM6B/JMJD3 inhibitor, for acute myeloid leukemia

**DOI:** 10.1007/s00432-018-2631-7

**Published:** 2018-03-28

**Authors:** Yunan Li, Mingying Zhang, Mengyao Sheng, Peng Zhang, Zizhen Chen, Wen Xing, Jie Bai, Tao Cheng, Feng-Chun Yang, Yuan Zhou

**Affiliations:** 1 State Key Laboratory of Experimental Hematology, Institute of Hematology and Blood Diseases Hospital, Chinese Academy of Medical Sciences and Peking Union Medical College, Tianjin, China; 2 Sylvester Comprehensive Cancer Center, University of Miami Miller School of Medicine, Miami, USA; 3 Department of Biochemistry and Molecular Biology, University of Miami Miller School of Medicine, Miami, USA; 4 Center for Stem Cell Medicine, Chinese Academy of Medical Sciences, Tianjin, China; 5 Department of Stem Cell and Regenerative Medicine, Peking Union Medical College, Tianjin, China; 6 Collaborative Innovation Center for Cancer Medicine, Tianjin, China

**Keywords:** Acute myeloid leukemia, KDM6B, Histone demethylase, Small molecule inhibitor

## Abstract

**Purpose:**

Acute myeloid leukemia (AML) is a heterogeneous disease with poor outcomes. Despite increased evidence shows that dysregulation of histone modification contributes to AML, specific drugs targeting key histone modulators are not applied in the clinical treatment of AML. Here, we investigated whether targeting KDM6B, the demethylase of tri-methylated histone H3 lysine 27 (H3K27me3), has a therapeutic potential for AML.

**Methods:**

A KDM6B-specific inhibitor, GSK-J4, was applied to treat the primary cells from AML patients and AML cell lines in vitro and in vivo. RNA-sequencing was performed to reveal the underlying mechanisms of inhibiting KDM6B for the treatment of AML.

**Results:**

Here we observed that the mRNA expression of *KDM6B* was up-regulated in AML and positively correlated with poor survival. Treatment with GSK-J4 increased the global level of H3K27me3 and reduced the proliferation and colony-forming ability of primary AML cells and AML cell lines. GSK-J4 treatment significantly induced cell apoptosis and cell-cycle arrest in Kasumi-1 cells, and displayed a synergistic effect with cytosine arabinoside. Notably, injection of GSK-J4 attenuated the disease progression in a human AML xenograft mouse model in vivo. Treatment with GSK-J4 predominantly resulted in down-regulation of DNA replication and cell-cycle-related pathways, as well as abrogated the expression of critical cancer-promoting *HOX* genes. ChIP-qPCR validated an increased enrichment of H3K27me3 in the transcription start sites of these *HOX* genes.

**Conclusions:**

In summary, our findings suggest that targeting KDM6B with GSK-J4 has a therapeutic potential for the treatment of AML.

**Electronic supplementary material:**

The online version of this article (10.1007/s00432-018-2631-7) contains supplementary material, which is available to authorized users.

## Introduction

Acute myeloid leukemia (AML) is characterized by clonal expansion of undifferentiated myeloid precursors (Cheng et al. 2014; Papaemmanuil et al. 2016). Although classical chemotherapy can achieve complete remission (CR) in the majority of AML patients, the relapse rate within 3 years of diagnosis is 50–70% and the 5-year overall survival in adult AML patients is only 5–55% (Al-Hussaini and DiPersio 2014; Byrd et al. 2002; Cheng et al. 2014). Novel therapeutic agents are desperately needed for AML to overcome continued poor outcomes. In the past 2 decades, whole-genome sequencing and other systematic studies of AML genetic landscapes revealed that many epigenetic modifiers are involved in AML initiation, progression and prognosis (Ryotokuji et al. 2016). Mutations of epigenetic modifiers alone or with other oncogenes contribute significantly to leukemogenesis (Nguyen et al. 2011; Tanaka et al. 2012).

Bhaumik et al. reported that alterations in histone H3 lysine (K) methylation are associated with aberrant expression of genes, which are critical for the cellular functions of hematopoietic stem cells and leukemic transformation (Bhaumik et al. 2007; Cui et al. 2009; Fiskus et al. 2014). Lysine-specific demethylase 6B (KDM6B), also known as Jumonji domain-containing protein D3 (JMJD3), is a member of the Jumonji C (JmjC) histone demethylase family, which are Fe^2+^ and α-ketoglutarate dependent (Mosammaparast and Shi 2010; Shi 2007). KDM6B removes the tri-methylation (me3) and di-methylation (me2) marks of histone H3 lysine 27 (H3K27) and results in activation of gene expression (Kruidenier et al. 2012). There is growing evidence that KDM6B is critical for multiple cellular functions, including differentiation of embryonic stem cells (ESCs) and mesenchymal stem cells (MSCs) (Jiang et al. 2013; Xu et al. 2013; Ye et al. 2012), inflammatory responses in macrophages, and cellular senescence by controlling of INK4 box and p53 (Kruidenier et al. 2012; Salminen et al. 2014). In addition, the expression of *KDM6B* is significantly elevated in specimen of multiple myeloma (MM) patients, in the bone marrow (BM) hematopoietic stem and progenitor cells (HSPCs) of patients with myelodysplastic syndrome (MDS) and chronic myelomonocytic leukemia (CMML) (Ohguchi et al. 2017; Oppermann 2016; Wei et al. 2016). In the current study, we sought to determine the effect of *KDM6B* expression in the outcome of AML patients and to test the impact of pharmacologic inhibition of KDM6B on AML. We found that *KDM6B* is overexpressed in patients with AML and these patients have a poor prognosis. KDM6B-specific pharmacological inhibitor, GSK-J4, dramatically induced anti-proliferative effects in AML cell lines and freshly isolated BM mononuclear cells (MNCs) from AML patients along with increasing levels of H3K27me3. GSK-J4 also resulted in cell apoptosis and cell-cycle arrest in vitro and reduced the tumor burden in an AML xenograft mouse model in vivo. Mechanistically, GSK-J4 treatment dramatically down-regulated DNA replication and cell-cycle-related pathways and the expression of *HOX* genes. In addition, the H3K27me3 enrichment in the transcription start sites (TSSs) of *HOXA5, HOXA7, HOXA9* and *HOXA11* was significantly increased, indicating the transcriptional suppression of these cancer-promoting genes. In summary, this study provides strong evidence for the potential of KDM6B as a novel therapeutic target in AML and, therefore, inhibiting KDM6B may serve as a novel treatment for AML.

## Materials and methods

### Primary AML cells and AML cell lines

Human BM samples were obtained from a cohort of 24 individuals with AML and five healthy volunteer donors from 2007 to 2016 at the Institute of Hematology and Blood Diseases Hospital, Chinese Academy of Medical Sciences. The sample collection procedures were in accordance with guidelines of the Helsinki Declaration, and written informed consents were obtained from all patients and healthy donors after approval of the Ethics Committee of our hospital. All patients were reevaluated and met the 2016 WHO diagnostic criteria for AML. BM mononuclear cells (MNCs) were isolated by standard Ficoll-Plaque density gradient separation procedure and frozen viably. The clinical characteristics of AML patients in this study were listed in Table S1 (Online Resource 1).

The AML cell lines which were used in this study were purchased from the American Type Culture Collection. Kasumi-1, a human AML cell line with t(8;21) translocation, was maintained in RPMI1640 (Gibco, Carlsbad, USA) with 20% fetal bovine serum (FBS, Gibco, Carlsbad, USA) and 1% penicillin/streptomycin (P/S) (Beyotime, Shanghai, China). THP-1, a human monocytic cell line derived from an acute monocytic leukemia patient, was cultured in RPMI1640 supplemented with 10% FBS and 1% P/S. KG-1 and KG-1a cells, two cell lines derived from a patient with AML, were cultured in IMDM supplemented with 20% FBS and 1% P/S. All cells were cultured at 37 °C in a 5% CO_2_ atmosphere.

### Cytotoxicity assay

MNCs from primary AML samples were seeded in 6-well plates at a concentration of 3 × 10^5^ cells/mL for 2 mL per well then treated with 5.5 μM GSK-J4 (Sigma-Aldrich, USA) or solvent control DMSO (Sigma-Aldrich, USA) for 24 h. Cells were then collected and counted by Trypan Blue (Sigma-Aldrich, USA) staining to determine the alive cell numbers. Cell survival after the 24-h drug treatment was calculated by the percentage of live cells in GSK-J4 treated group divided by that in DMSO group for every single sample. For leukemia cell lines, we seeded Kasumi-1, THP-1, KG-1 and KG-1a cells in 96-well plates (3 × 10^4^ cells/well). After a 72-h treatment of GSK-J4 with various concentrations, Cell Counting Kit-8 (CCK-8, Dojindo Laboratories, Japan) solution was added and then incubated at 37 °C in a 5% CO_2_ incubator for additional 4 h. Absorbance was measured using Micro-plate Reader (Synergy H4, BioTek, USA) at the absorbance of 450 nm. The data was graphically displayed and the IC_50_ was determined using GraphPad Prism 5 software (GraphPad Software, USA).

### Colony-forming cell assay

Primary MNCs of AML patients or leukemic cell lines were cultured in the presence or absence of 5.5 μM GSK-J4 for 24 h, harvested and washed twice with PBS. Then the treated and untreated primary MNCs, or AML cell lines, were re-counted and plated into 24-well plates in 0.5 mL methylcellulose medium (MethoCult H4434, StemCell Technologies, Canada) at cell densities of 2 × 10^5^ cells/mL or 1 × 10^3^ cells/mL, respectively. Cells were cultured for 14 days then the types and numbers of colonies were identified and numerated under a microscope.

### Cell growth, apoptosis and cell-cycle analyses

Kasumi-1 cells were cultured in 24-well plates with or without GSK-J4 for 7 days to test the effect of GSK-J4 on cell growth. Each condition of the cultures was triplicated at each time point and cell numbers were counted manually every day. Kasumi-1 cells were collected and treated with 5.5 μM GSK-J4 or solvent control (DMSO) for 24 h to determine if GSK-J4 affect apoptosis of AML cells. Apoptosis assay was performed with JC-1 staining (AAT Bioquest, Sunnyvale, CA, USA) to detect the mitochondrial membrane potential (MMP) according to the manufacturer’s protocol. JC-1 is capable of selectively entering mitochondria, and reversibly forming JC-1 aggregates upon mitochondria membrane polarization that causes shifts in emitted light from green (emission of JC-1 monomeric form, detected through the FITC channel) to red (emission of JC-1 aggregate form, detected through PE channel). Apoptotic cells demonstrate a decrease in MMP and the increased emitted green fluorescence can be detected by flow cytometry. To determine the impact of GSK-J4 on cell cycle, Kasumi-1 cells were treated by 5.5 μM GSK-J4 for 24 h, followed by BrdU and 7-AAD (BrdU Flow Kits, BD Biosciences, USA) double staining. The percentage of cells in different cell-cycle status was determined by flow cytometry.

### Combination index determination

The combination index (CI) values for GSK-J4 and cytosine arabinoside (Ara-C) (Pfizer, New York, USA) were calculated by median dose-effect analysis (assuming mutual exclusivity) using CalcuSyn Version 2.1 (Biosoft, Ferguson, MO) software. CI values less than 1.0 represent a synergistic interaction of the combination treatment (Fiskus et al. 2014; Kojima et al. 2008).

### Chromatin immunoprecipitation (ChIP)-qPCR

The chromatin immunoprecipitation (ChIP) assay was performed with EZ-Magna ChIP™ A-Chromatin Immunoprecipitation Kit (Millipore, Temecula, USA) according to the manufacturer’s recommendations. Gene expression was measured by real-time quantitative PCR (qPCR) as we previously described (Si et al. 2016). The primers used in qPCR were shown in Table S2 and S3 (Online Resource 1).

### Western blot assay

Whole-cell lysates of GSK-J4 or DMSO treated cells were extracted by SDS loading buffer (Beyotime, China) and subjected to western blot analyses. The antibodies used for western blot include H3 (ab1791, Abcam, England) and H3K27me3 (C36B11, Cell Signaling Technology, USA). These two antibodies along with normal rabbit IgG (2729, Cell Signaling Technology, USA) were also used in ChIP assays. All the experiments were repeated at least twice with similar results.

### RNA-sequencing and bioinformatic analyses

RNA-sequencing experiments for Kasumi-1 cells treated with DMSO or 5.5 µM GSK-J4 were performed by Novogene (Beijing, China). Briefly, total RNAs were isolated from Kasumi-1 cells (three biological replicates per condition) using TRIzol reagent (Life Technologies, USA), and the RNA qualifications were confirmed by 1% agarose gels and the Bioanalyzer 2100 system (Agilent Technologies, CA, USA). Sequencing libraries were generated using NEBNext^®^ Ultra™ RNA Library Prep Kit for Illumina^®^ (NEB, USA) following manufacturer’s protocols. After cluster generation, the libraries were sequenced on an Illumina Hiseq platform, and 150 bp paired-end reads were generated. The split read aligner TopHat (v2.0.12) and Bowtie2 (v2.2.3) were used to align the reads to the appropriate genomes. HTSeq (v0.6.1) was used to count the read numbers mapped to each gene and FPKM method was used for determining gene expression levels. Differential expression analysis of GSK-J4 and DMSO groups was performed using the DESeq R package (1.18.0). Genes with an adjusted *p* value < 0.05 and fold change > 1.6 found by DESeq were assigned as differentially expressed. Gene Ontology (GO) enrichment analysis of differentially expressed genes was implemented by the GOseq R package and the KOBAS software was used to test the statistical enrichment of differentially expressed genes in KEGG pathways.

### Mouse model of human AML

To validate the anti-leukemic effect of GSK-J4 in vivo, 1 × 10^7^ Kasumi-1 cells were injected via tail vein into 5-week-old female NOD-Prkdc^em26Cd52^Il2rg^em26Cd22^/Nju (NCG) mice (Nanjing Biomedical Research Institute of Nanjing University, China) following a sub-lethal irradiation at a dose of 200 cGy. GSK-J4 (50 mg/kg) or solvent control (1.5% DMSO in 0.9% saline) was administered into the recipient mice via peritoneal injection once a day for 4–6 weeks (5-day injection and 2-day break for each week). The final concentration of diluted GSK-J4 was 5 mg/mL and the injection volume was ~ 300 μL. Mice were humanely killed in accordance with Institutional Animal Care and Use Committee (IACUC) protocols at 10 weeks. BM cells (mixed cells from tibias and femurs) were flushed and suspended in PBS for phenotypic analysis by flow cytometry. BM, spleens, livers and kidneys were fixed in 10% Accustain Formalin Solution (Sigma-Aldrich, USA) and embedded in paraffin for hematoxylin and eosin (H&E) staining. All animal experiments were approved by the Animal Research Committee of our hospital.

### Statistical analysis

BloodSpot database was used to retrieve mRNA expression data for the expression patterns of *KDM6B* in AML patients and normal hematopoietic stem cells (HSCs) (http://servers.binf.ku.dk/bloodspot/) (Bagger et al. 2016). The relationship between *KDM6B* gene expression and prognosis in patients with AML was examined using the PrognoScan database (http://www.abren.net/PrognoScan/) (Mizuno et al. 2009). The patient samples were split into high and low *KDM6B* expression groups with the optimal cutoff point determined by the minimum *p* value approach. The *p* value was calculated by the log-rank test and corrected according to the database description.

Experimental data was reported as mean with standard error of the mean (SEM), unless otherwise indicated. Unpaired *t* test or Mann–Whitney test was used for two group comparisons, and ANOVA analysis was used to determine differences among three or more groups. A *p* value of < 0.05 was considered significant.

## Results

### The expression of *KDM6B* is up-regulated in AML

**Fig. 1:**
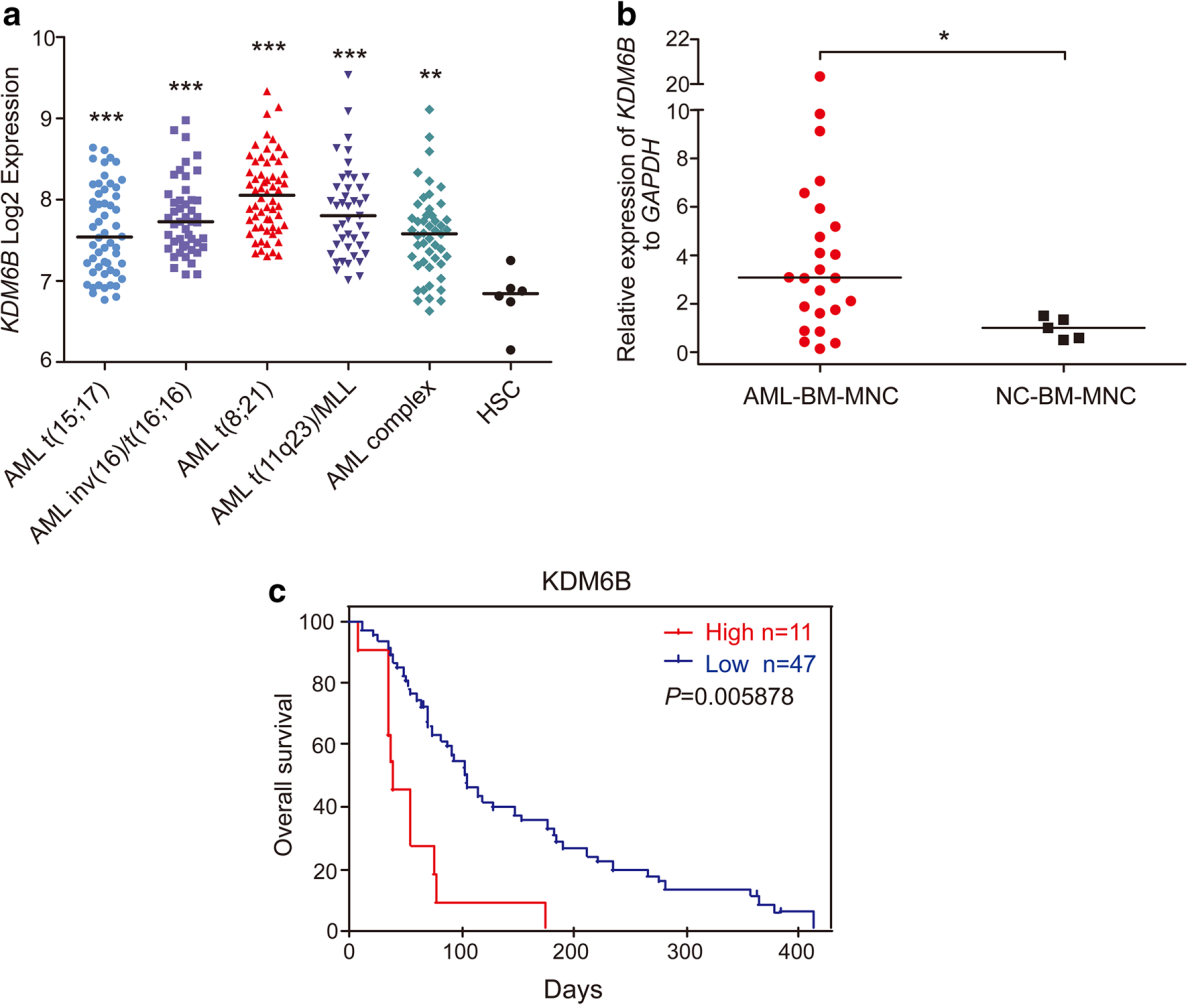
*KDM6B* is up-regulated in human AML. **a** Analysis of *KDM6B* mRNA expression levels in primary AML samples with t(15;17), inv(16)/t(16;16), t(8;21), t(11q23) translocations, AML complex and normal human HSCs (Lin^−^ CD34^+^ CD38^−^ CD90^+^ CD45RA^−^) using the Bloodspot database. The horizontal bar indicates the median value of each group. **p < 0.01, ***p < 0.001, Mann–Whitney *U* test. **b** Comparison of *KDM6B* mRNA expression in bone marrow (BM) mononuclear cells (MNCs) between AML patients (*n* = 24) and healthy controls (*n* = 5, described as normal control). The expression levels of *KDM6B* were normalized to *GAPDH*. The horizontal bar indicates the median value of each group. *p < 0.05, Mann–Whitney *U* test. **c** Kaplan–Meier analysis showing the AML patients with high expression levels of *KDM6B* (red, *n* = 11) had a significant shorter overall survival rate than patients with low expression levels (blue, *n* = 47). **p < 0.01, Meta-analyses of microarray data are from the PrognoScan database

Gene expression profiles (GSE42519 and GSE13159) for normal and malignant hematopoietic cells were analyzed with Bloodspot database to determine the mRNA expression of *KDM6B* in AML. A cohort of AML samples (*n* = 252) were included in this analysis. When compared to normal hematopoietic stem cells (HSCs), the expression levels of *KDM6B* mRNA were substantially higher in each type of AML samples, including AML with t(15;17) (*n* = 54), AML with inv(16)/t(16;16) (*n* = 47), AML with t(8;21) (*n* = 60), AML with t(11q23) (*n* = 43) and AML complex (*n* = 48), and highest *KDM6B* overexpression was observed in AML with t(8;21) translocation (Fig. 1a). Moreover, we measured the *KDM6B* mRNA expression in BM samples from AML patients (*n* = 24) and healthy donors (*n* = 5, described as normal control) collected from our hospital. In line with the Bloodspot results, *KDM6B* was highly expressed in AML BM MNCs than in normal controls (Fig. 1b). A PrognoScan database-based Kaplan–Meier analysis of the overall survival of 58 AML patients by high (*n* = 11) and low (*n* = 47) *KDM6B* levels revealed that high *KDM6B* expression positively correlated with poor overall survival in human AML (data from GSE5122) (Fig. 1c). Collectively, all these results suggest that the evaluated expression of *KDM6B* may be a potential target in AML.

### GSK-J4 inhibits the proliferation of AML cells

**Fig. 2:**
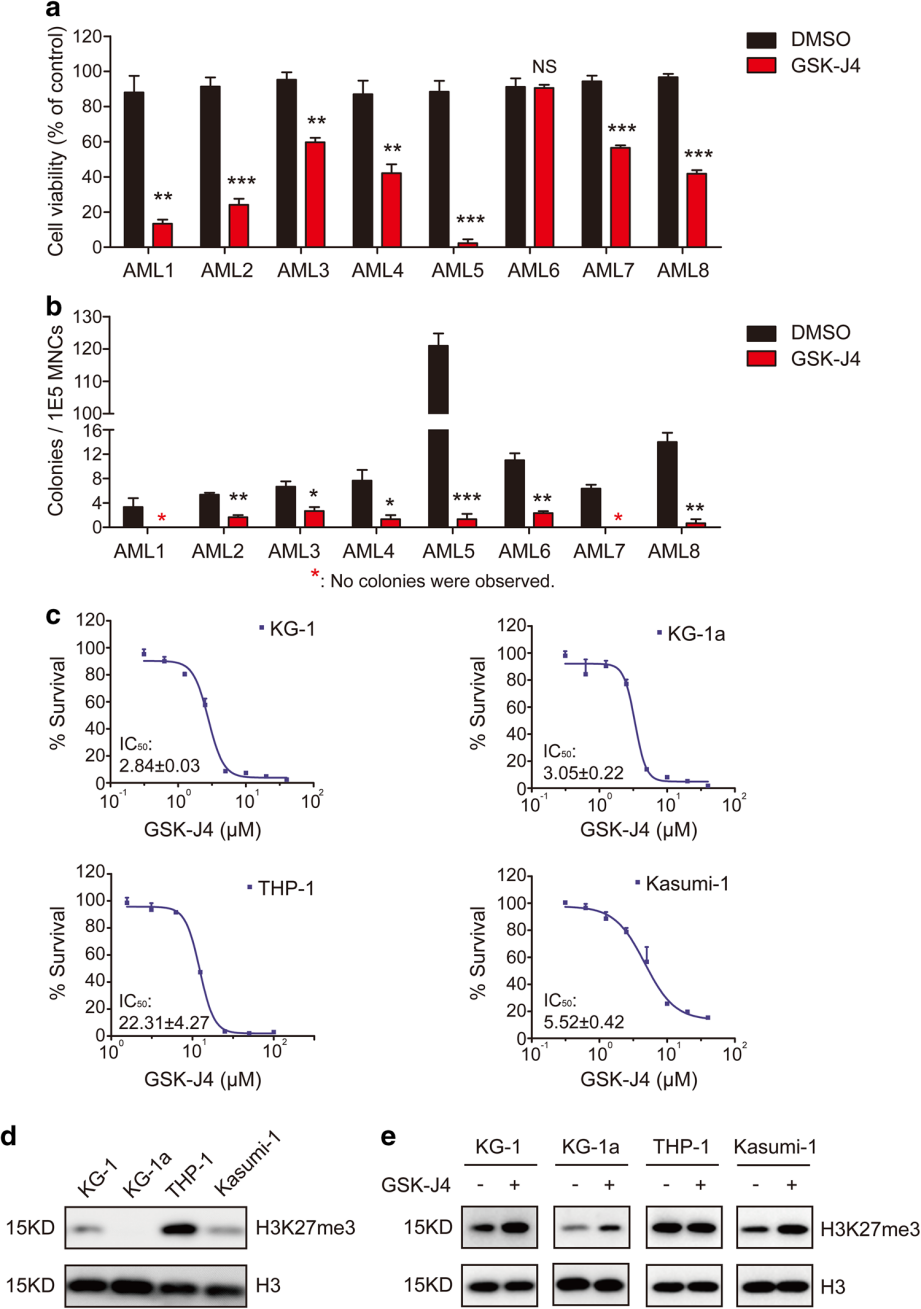
GSK-J4 treatment inhibits the proliferation of AML cells. **a** Primary human AML MNCs were treated with 5.5 µM GSK-J4 or corresponding content of DMSO for 24 h. Cell numbers were counted by Trypan Blue exclusion assay and normalized with the control group. Data represents mean ± SEM. **p < 0.01, ***p < 0.001, *NS* no significance, two-tailed Student *t* test. **b** Total colony numbers of primary AML MNCs with 5.5 μM GSK-J4 treatment were counted after 14-day methylcellulose culture. The red asterisk represents that no colonies were observed. Data represents mean ± SEM. *p < 0.05, **p < 0.01, ***p < 0.001, two-tailed Student *t* test. **c** Cytotoxic analysis of GSK-J4 in different human AML cell lines. Cytotoxicity was determined by CCK-8 assay and displayed as inhibition curves with IC_50_ values. KG-1, KG-1a, THP-1 and Kasumi-1 cells were treated with a range dose of GSK-J4 for 72 h, respectively. Numerical analysis data represents mean ± SEM. **d** Western blot showed the H3K27me3 levels in KG-1, KG-1a, THP-1 and Kasumi-1 cells. **e** Globally increased H3K27me3 levels were observed in KG-1, KG-1a and Kasumi-1 cells after 72-h treatment of 5.5 μM GSK-J4

Next, we evaluated the effects of GSK-J4, a small molecule inhibitor of KDM6B, on the growth of AML primary cells and cell lines. In 7 out of 8 primary AML samples, the percentage of survival rates was reduced remarkably after culturing with 5.5 μM GSK-J4 for 24 h, in comparison to the group treated with DMSO (Fig. 2a). In the semisolid culturing, the addition of GSK-J4 significantly decreased the number of colony-forming units (CFUs) derived from AML MNCs compared with vehicle control (Fig. 2b; Fig. S1a in Online Resource 1). After 14-day of colony forming, the percentage of viable cells in GSK-J4 treated plates was significantly decreased (Fig. S1b in Online Resource 1). It has been reported that GSK-J4 inhibited the KDM6B-induced loss of nuclear H3K27me3 in HeLa cells and T-ALL cells (Kruidenier et al. 2012; Ntziachristos et al. 2014). Following GSK-J4 treatment, we also observed an increased level of H3K27me3 in primary AML cells (Fig. S1c in Online Resource 1).

To evaluate the half-maximal inhibitory concentrations (IC_50_) of GSK-J4 in different AML cells, several leukemia cell lines, including Kasumi-1, KG-1, KG-1a and THP-1, were cultured at a range of concentrations of GSK-J4 for 72 h, and the percentage of cells that had survived was evaluated using a CCK-8 kit. GSK-J4 had shown significant inhibitory effects on KG-1, KG-1a and Kasumi-1 cells with IC_50_ values of 2.84, 3.05 and 5.52 μM, respectively (Fig. 2c). In contrast, the IC_50_ value of THP-1 cells exceeded 20 µM (Fig. 2c). Since the H3K27me3 expression levels of these cell lines differed dramatically according to our western blot determination (Fig. 2d), the drug sensitivity discrepancy between THP-1 and other three AML cell lines may result from the high background level of H3K27me3 in THP-1 cells (Fig. 2d). Consistent with the results from primary AML cells, GSK-J4 treatment significantly increased the H3K27me3 levels in KG-1, KG-1a and Kasumi-1 (Fig. 2e). These data indicates that GSK-J4 treatment may affect the proliferation of primary AML MNCs and leukemic cell lines by increasing the levels of repressive H3K27me3 mark.

### GSK-J4 induces apoptosis and cell-cycle arrest and has a synergistic effect with Ara-C in Kasumi-1 cells

**Fig. 3:**
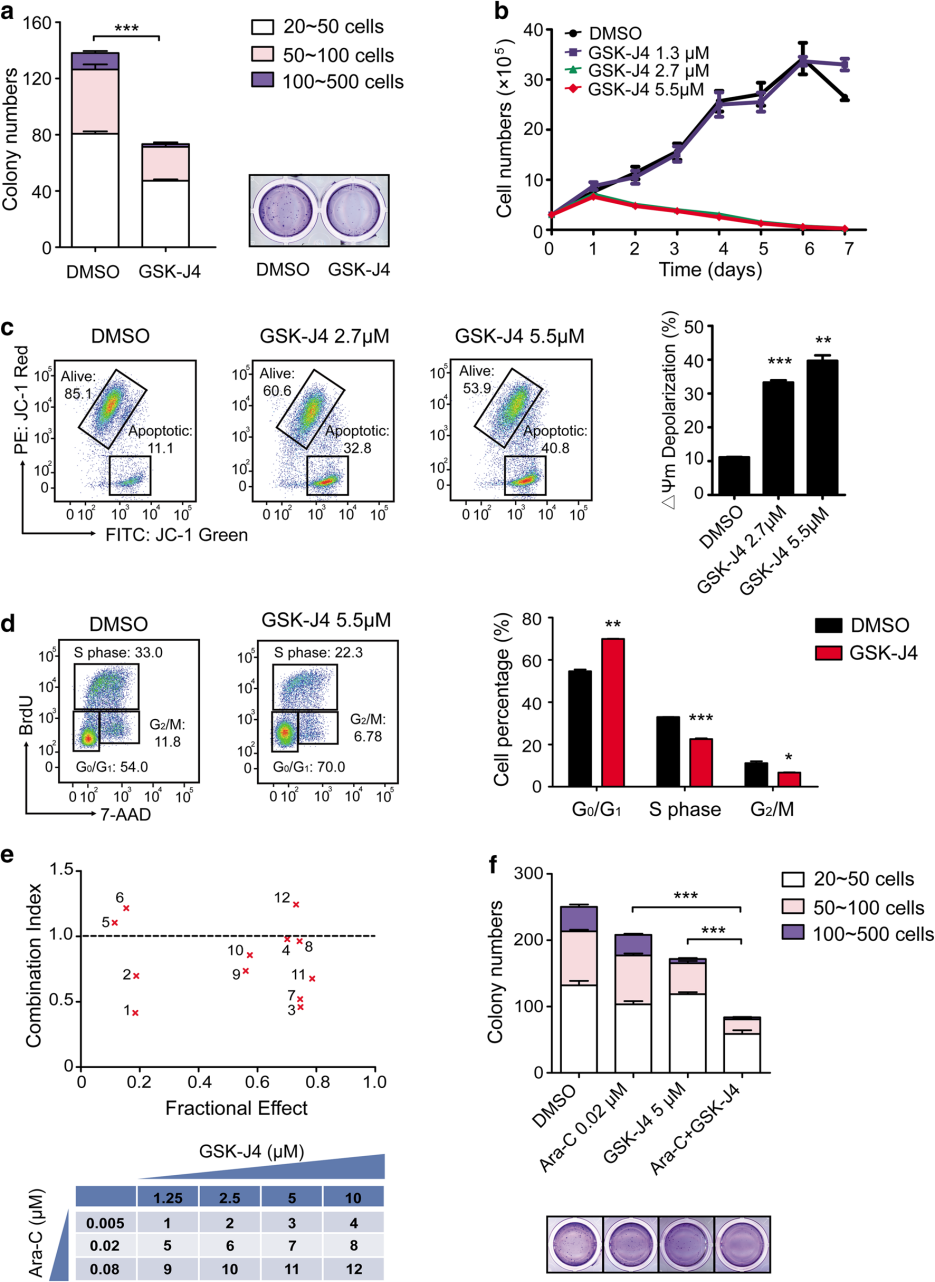
GSK-J4 inhibits the proliferation and colony formation of Kasumi-1 cells by inducing apoptosis and cell-cycle arrest. **a** The number and size of colonies decreased in Kasumi-1 cells treated with GSK-J4. ***p < 0.001, two-tailed Student *t* test. **b** Growth curve of Kasumi-1 cells treated with GSK-J4 at indicated concentrations. Error bars indicate the SEM of triplicates. **c** Dose-dependent apoptosis was induced by GSK-J4 in Kasumi-1 cells, as determined by the specific fluorescent probe of mitochondrial membrane potential (MMP), JC-1. In Kasumi-1 cells treated with GSK-J4, increase in the emitted light of JC-1 Green (FITC channel) and decrease in JC-1 Red (PE channel) indicate depolarization of mitochondria during apoptosis, as compared to DMSO treated cells. Data represents mean ± SEM, **p < 0.01, ***p < 0.001, two-tailed Student *t* test. **d** Cell-cycle arrest was induced by GSK-J4 in Kasumi-1 cells. After a 24-h GSK-J4 treatment, the percentage of cells at G_0_/G_1_ phase increased while the S phase and G_2_/M phase decreased significantly. Data represents mean ± SEM, *p < 0.05, **p < 0.01, ***p < 0.001, two-tailed Student *t* test. **e** GSK-J4 synergized with Ara-C to inhibit proliferation and colony-forming capability of Kasumi-1 cells in vitro. Combination Index (CI) versus Fractional Effect (FE) plot was derived from CalcuSyn Software and CI values < 1.0 indicate synergy, whereas CI values > 1.0 indicate antagonism. The following table described the dose combinations shown on the CI versus FE plot. **f** The colony numbers of Kasumi-1 cells treated with GSK-J4, Ara-C and a combination of these two drugs were shown. Data represents mean ± SEM, ***p < 0.001, two-tailed Student *t* test

Having established that the expression of *KDM6B* was clearly the highest in t(8;21) AML patients amongst all the AML subtypes (Fig. 1a) and Kasumi-1 is an AML cell line with *AML1-ETO* fusion gene (Asou et al. 1991), we then focused the following studies with Kasumi-1 to investigate the effects of GSK-J4 on leukemic cells in vitro and in vivo. Similar to the results obtained from the primary AML cells, GSK-J4 treatment significantly impaired the colony formation ability of Kasumi-1 cells (Fig. 3a). GSK-J4 at the concentration of 5.5 μM (IC_50_) or 2.7 μM inhibited the growth of Kasumi-1 cells in liquid cultures in a time-dependent manner (Fig. 3b). Notably, we also observed that the addition of GSK-J4 significantly decreased mitochondrial membrane potential of Kasumi-1 cells in a dose-dependent manner, indicating the induction of apoptosis (Fig. 3c).

Then we performed flow cytometric analysis following BrdU and 7-AAD labeling to confirm the effect of GSK-J4 on cell cycle in leukemic cells. After a 24-h culture with 5.5 μM GSK-J4, the percentage of Kasumi-1 cells at G_0_/G_1_ phase had significantly increased. In contrast, the percentage of Kasumi-1 cells at S phase and G_2_/M phase had declined significantly (Fig. 3d). GSK-J4 also displayed a synergistic effect with cytosine arabinoside (Ara-C), one of the most commonly used anti-leukemic agent, on inhibiting cell proliferation as shown by the combination index (CI) versus Fractional Effect (FE) plot (Fig. 3e), and on reducing the number of CFUs (Fig. 3f). Accumulatively, these results demonstrate that GSK-J4 suppresses the proliferation and colony-forming capacity of Kasumi-1 cells alone or in combination with Ara-C, as well as increases apoptosis and induces cell-cycle arrest.

### GSK-J4 reduces tumor burden in an AML xenograft mouse model in vivo

**Fig. 4:**
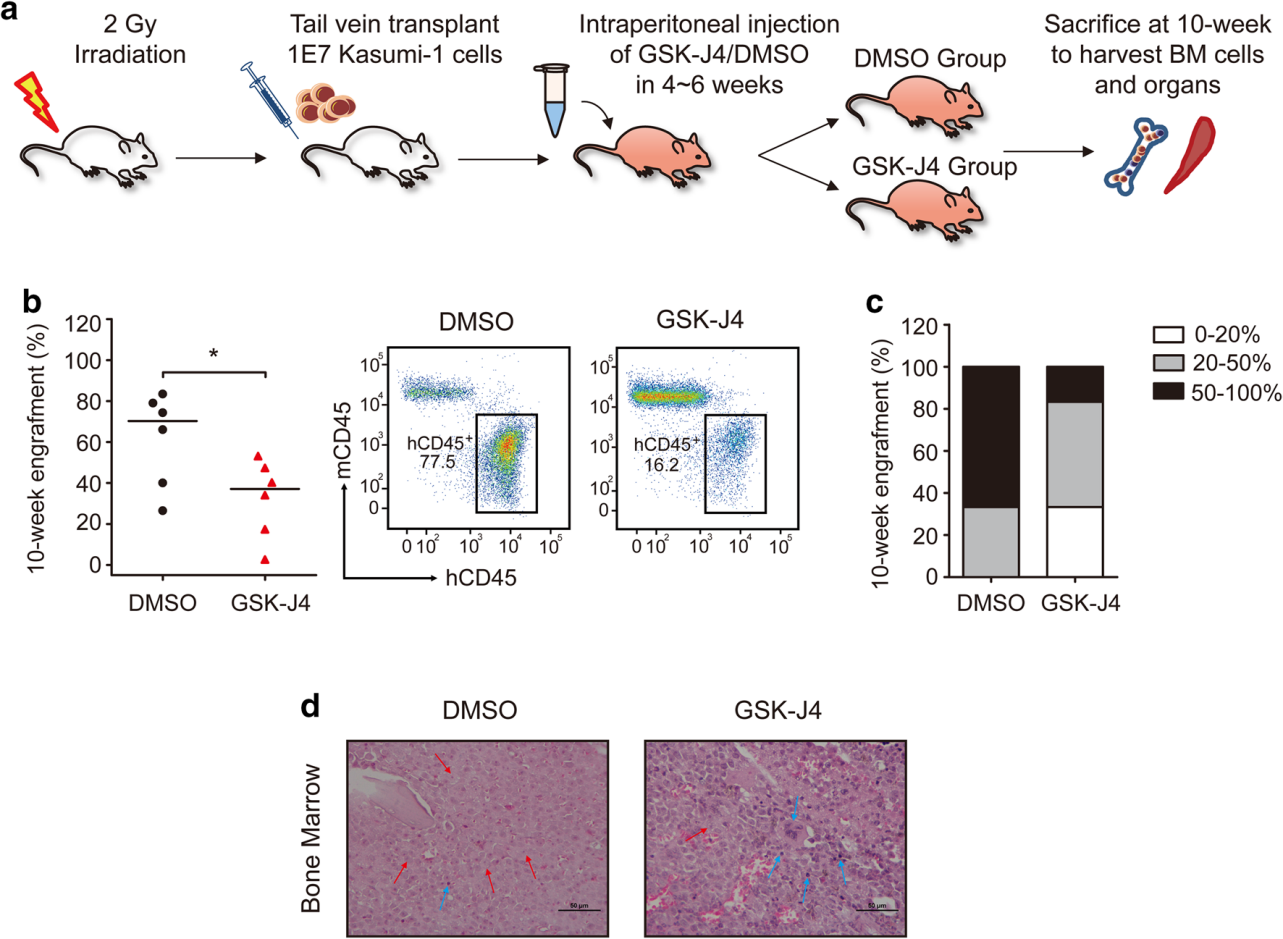
GSK-J4 reduces tumor burden in the Kasumi-1 cells transplanted xenograft mouse model in vivo. **a** Schema depicted the strategy of NCG mice transplantation with human Kasumi-1 cells. GSK-J4 was applied at a dose of 50 mg/kg during 4–6 weeks by intraperitoneal injection. **b** The percentage of hCD45^+^ mCD45^−^ human leukemic cells in the BM of mice was analyzed 4 weeks after GSK-J4 or DMSO treatment (week 10). The horizontal bar indicates the median value of each group. *p < 0.05, two-tailed Student *t* test. Representative scatter plots of flow cytometry showed the engrafted human leukemic cell populations after DMSO or GSK-J4 treatment. **c** The proportions of hCD45^+^ mCD45^−^ cell engraftments in the BM were divided into three groups (0–20%, 20–50% and 50–100%) after mice treated with DMSO or GSK-J4. **d** Representative photographs of histological BM sections by H&E staining from mice treated with DMSO or GSK-J4 were shown. Scale bars represent 50 μm. Lightly stained, small nuclei cells with rich cytoplasm are human leukemia cells (red arrows), while the deeply stained, relatively smaller cells are normal mouse hematopoietic cells (blue arrows)

To detect the effect of GSK-J4 on leukemic cell growth in vivo, we established an AML xenograft mouse model by transplanting Kasumi-1 cells into sub-lethally irradiated NCG mice (Fig. 4a). GSK-J4 treatment significantly decreased the engraftment of hCD45^+^ mCD45^−^ human leukemic cells in mice BM compared with vehicle control group (Fig. 4b). Moreover, the percentage of mice with high leukemia infiltration (proportion of hCD45^+^ mCD45^−^ cells > 50%) was lower in GSK-J4 treated group (Fig. 4c). Histologic analysis of the H&E stained femur sections revealed that GSK-J4 treated mice had more normal architectures and less human leukemia cells in BM (Fig. 4d). However, no leukemic infiltration was observed in the spleens of GSK-J4 and the vehicle control treated mice (Fig. S2 in Online Resource 1). Importantly, GSK-J4 treatment did not induce apparent toxicity to livers and kidneys of NCG mice (Fig. S2 in Online Resource 1). This in vivo study suggests that GSK-J4 reduces the leukemia burden in a Kasumi-1 xenograft mouse model, providing evidence for the potential application of GSK-J4 in leukemia treatment clinically.

### GSK-J4 performs its anti-leukemia effects by regulating cell cycle and histone modification

**Fig. 5:**
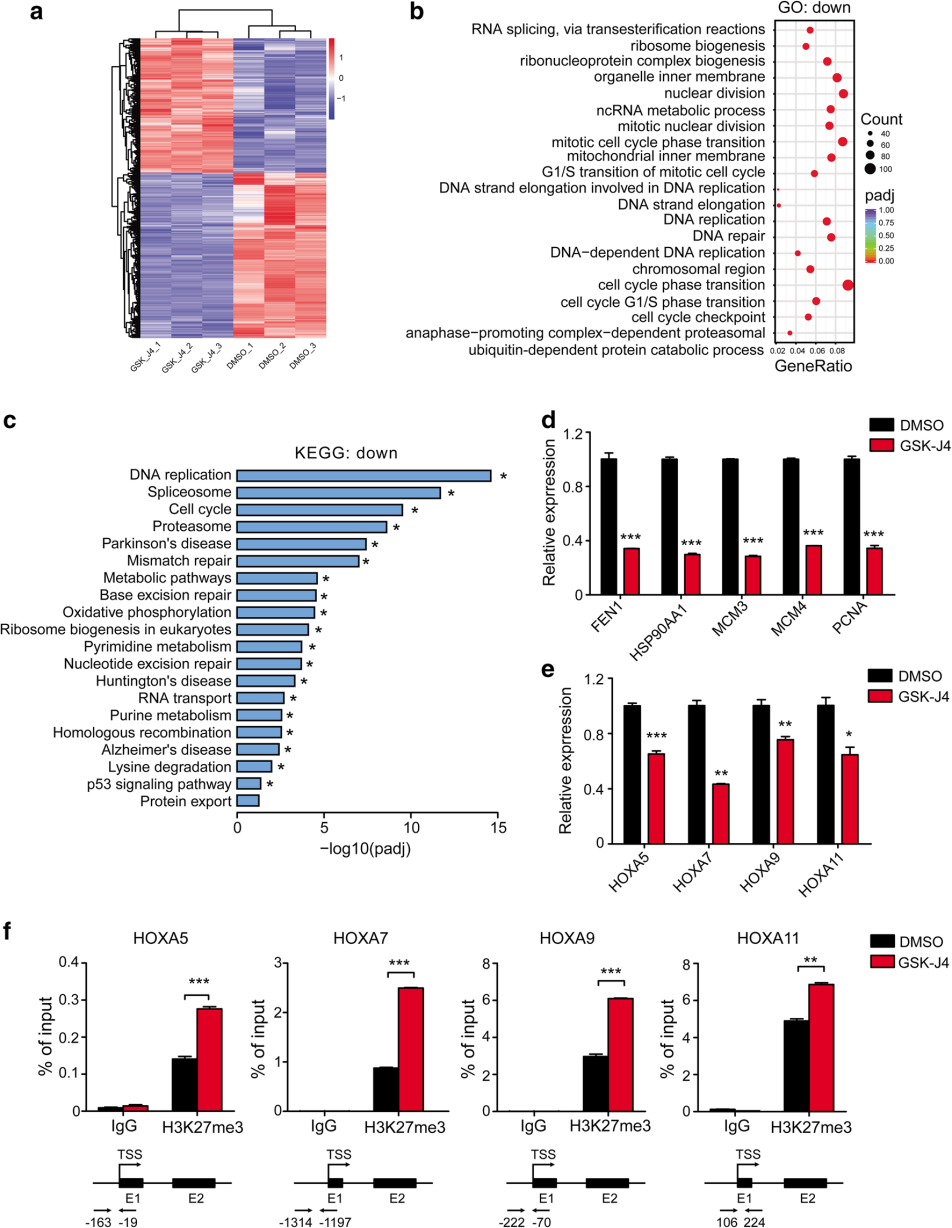
GSK-J4 treatment alters the transcriptional profiles of Kasumi-1 cells. **a** Heatmap of the significantly differentially expressed genes between DMSO and GSK-J4 treated Kasumi-1 cells (*n* = 3 per group, adjusted *p* < 0.05, fold change > 1.6). **b** Dot plots showed the top-20 significantly enriched GO terms for down-regulated genes (adjusted *p* < 0.05). **c** Histogram of top-20 significantly enriched KEGG pathways for down-regulated genes (adjusted *p* < 0.05). **d** The expression levels of key down-regulated genes related to DNA replication and cell-cycle progression in Kasumi-1 cells treated with GSK-J4 were verified by qPCR. Gene expression was normalized to *GAPDH*. ***p < 0.001, two-tailed Student *t* test. **e** *HOX* genes were down-regulated in Kasumi-1 cells treated with GSK-J4. *p < 0.05, **p < 0.01, ***p < 0.001, two-tailed Student *t* test. **f** ChIP-qPCR revealed the increased H3K27me3 enrichment in the promoter regions of *HOXA5, HOXA7, HOXA9* and *HOXA11* genes in GSK-J4 treated Kasumi-1 cells. **p < 0.01, ***p < 0.001, two-tailed Student *t* test

To identify the molecular mechanisms underlying the inhibitory effects of GSK-J4 on AML cells, we performed the RNA-seq analysis in Kasumi-1 cells treated with either GSK-J4 or DMSO control (Fig. 5a). Differentially expressed genes (DEGs) analysis showed 1375 up-regulated genes and 1354 down-regulated genes in GSK-J4 treated group compared with the DMSO group (adjusted *p* value < 0.05 and fold change > 1.6), and heatmap of the top-20 significantly differentially expressed genes between GSK-J4 and DMSO treated Kasumi-1 cells were listed (Fig. S3a in Online Resource 1). Gene Ontology (GO) and KEGG pathway analyses revealed that the down-regulated GO terms and KEGG pathways of GSK-J4 group far surpassed the up-regulated terms (516 down-regulated versus 2 up-regulated for GO analysis, 19 down-regulated versus none up-regulated for KEGG analysis, screened by adjusted *p* value < 0.05) (Fig. 5b, c), consistent with the increase in the levels of repressive H3K27me3 mark following GSK-J4 treatment. The most down-regulated GO term mediated by GSK-J4 treatment was cell-cycle phase transition, and the most down-regulated KEGG pathway was DNA replication, both are consistent with the cell-cycle arrest and growth inhibition observed in our study (Fig. 5b, c). Down-regulated genes related to RNA splicing or spliceosome were also significantly enriched in GSK-J4 treated group (Fig. 5b, c). In addition, the most significant up-regulated GO term was an inflammatory response in keeping with a previous report (Fig. S3b in Online Resource 1) (Kruidenier et al. 2012), and genes controlling cell differentiation and apoptosis were also up-regulated in GSK-J4 treated cells (Fig. S3a, c in Online Resource 1).

Furthermore, we performed quantitative real-time PCR (qPCR) on several key target genes that were most down-regulated in GSK-J4 treated group to validate the results of RNA-seq. As shown in Fig. 5d, the expression of *FEN1, HSP90AA1, MCM3, MCM4* and *PCNA*, which were known to regulate DNA replication and cell cycle, were significantly decreased in Kasumi-1 cells treated with GSK-J4 (Alabert and Groth 2012; de Zoeten et al. 2011; Groth et al. 2007). GSK-J4 treatment also resulted in a significant down-regulation of *HOXA5, HOXA7, HOXA9* and *HOXA11* expression in Kasumi-1 cells (Fig. 5e), whose transcription were regulated by H3K27me3 (Agger et al. 2007). We then performed ChIP-qPCR to determine if the reduction of *HOX* genes was associated with the changes of H3K27me3 induced by GSK-J4 treatment. A significant increase in H3K27me3 enrichment was observed around the TSS regions of *HOXA5, HOXA7, HOXA9* and *HOXA11* (Fig. 5f). Collectively, these results suggest that the anti-leukemia effects of GSK-J4 may due to the up-regulation of H3K27me3, regulating the expression of genes controlling DNA replication and cell cycle as well as cancer-promoting *HOX* genes, directly or indirectly.

## Discussion

Regulation of histone methylation/demethylation was capable of affecting a wide range of important biological and pathological processes (Audia and Campbell 2016; Vastenhouw and Schier 2012). H3K27me3 is a pivotal repressive post-transcriptional regulation mark which was controlled by Polycomb repressive complex 2 (PRC2) and histone demethylase KDM6B and KDM6A. It has been reported that *KDM6B* is highly expressed in T cell acute lymphoblastic leukemia (T-ALL) and is essential for its initiation and maintenance (Ntziachristos et al. 2014). The overexpression of *KDM6B* has also been observed in MDS BM CD34^+^ cells (Wei et al. 2013). In this paper, we reported that in AML samples, *KDM6B* mRNA expression was significantly higher than normal controls based on the analysis of data from our lab and Bloodspot database.

There are emerging evidences showing that dysregulation of H3K27me3 was involved in the pathogenesis of several hematopoietic malignancies including AML (Shih et al. 2012; Ueda et al. 2016). The fact that the transcriptional activation of downstream oncogenes can result from the loss of repressive H3K27me3 mark, led us to hypothesize that the ectopic expression of histone demethylase such as *KDM6B* may be a reasonable therapeutic target in AML.

When considering the inherent reversibility of epigenetic marks, disordered epigenetic regulation poses the chance to use targeted drugs. Up until now, small molecule inhibitors targeting histone modifiers have made some progress in hematopoietic malignancies (Bots et al. 2014; Fiskus et al. 2009; Lubbert and Kuendgen 2015). For example, inhibitors of histone H3K79 methyltransferase DOT1L selectively inhibited the proliferation of MLL-rearranged cells by inducing cell differentiation and apoptosis (Daigle et al. 2011); histone demethylase LSD1 inhibitors can rescue the blocked AML cell differentiation and inhibit the colony growth of AML cells (Fiskus et al. 2014; Ishikawa et al. 2017; Sugino et al. 2017). The potent H3K27me3 demethylase inhibitor, GSK-J4, was used for regulating the immune response in human primary macrophages firstly (Kruidenier et al. 2012). Thanks to the growing studies of KDM6B, GSK-J4 has been applied to different kinds of tumors such as T-ALL, diffuse large B-cell lymphoma, pediatric brainstem glioma and prostate cancer with dismal toxicity for normal hematopoietic progenitor cells (Hashizume et al. 2014; Mathur et al. 2017; Morozov et al. 2017; Ntziachristos et al. 2014). A most recent study by Boila et al. also reported the application of GSK-J4 in AML (Boila et al. 2017). In addition to their report, we found that GSK-J4 demonstrated effective inhibition of cell survival and cell-cycle progression in Kasumi-1, an AML cell line with t(8;21) translocation, and KG-1/KG-1a, two primitive myeloid leukemic cell lines with high CD34 expression levels. Since AML samples with t(8;21) translocation showed the highest expression level than other most common translocations based on the analysis of 252 samples from Bloodspot database, a xenograft model by transplanting Kasumi-1 cells into sub-lethally irradiated NCG mice was applied to testify the effects of GSK-J4 on AML cells in vivo. Our data demonstrated that the application of GSK-J4 led to reduced tumor burden and leukemic infiltration in the BM of mice, indicating the therapeutic potential of GSK-J4 to AML in vivo.

In our study, we also found that GSK-J4 showed a synergistic effect in combination with Ara-C, a first line chemotherapeutic drug in AML, to inhibit colony forming corporately in AML cells, which is consistent with the report that application of GSK-J4 can sensitize diffuse large B-cell lymphoma to chemotherapeutic drugs such as Doxorubicin, Bortezomib and Vorinostat by inducing cell apoptosis (Mathur et al. 2017). However, the synergism of GSK-J4 with other commonly used drugs in AML still needs to be further explored.

Importantly, here we reported the potential mechanisms of the therapeutic effects of GSK-J4 in AML by RNA-sequencing. We found that the predominantly effect of GSK-J4 treatment in Kasumi-1 cells was downregulating some critical genes related to cell-cycle phase transition and DNA replication such as *FEN1, HSP90AA1, MCM3, MCM4* and *PCNA*, which was consistent with the cellular changes in GSK-J4 treated Kasumi-1 cells. It has been reported that FEN-1 and PCNA are two central components of a complex that generating DNA ligation products (Chapados et al. 2004). MCM3 and MCM4 are subunits of the hexameric MCM2-7 complex required for the initiation and elongation of DNA replication in all eukaryotes (Lei et al. 2002). The decreased expression of *FEN-1, PCNA, MCM3* and *MCM4*, together with the key cell-cycle-regulated gene *HSP90AA1*, corporately contributed to the antineoplastic effect of GSK-J4 in Kasumi-1 cells through regulating DNA replication and cell cycle. In addition, the mRNA expression of *CDKN2A* (*P16-INK4A*) was also significantly decreased in GSK-J4 treated Kasumi-1 cells, which is consistent with previous report that KDM6B can target INK4A in human papillomavirus E7 cells (McLaughlin-Drubin et al. 2011). Notably, some of the differentially expressed genes following GSK-J4 treatment were also enriched in RNA splicing and spliceosome, such as *SF3B5* and *SRSF1* (data not shown), suggesting that the mechanisms underlying GSK-J4 mediated effects on AML cells may be more complex.

*HOX* genes, such as *HOXA7* and *HOXA9*, are essential for the transformation of myeloid progenitors into leukemic cells with leukemogenic fusion proteins (Ayton and Cleary 2003). High *HOXA5* expression is also associated with low complete remission rate in AML (Zhao et al. 2015). In our study, we demonstrated that the mRNA levels of *HOXA5, HOXA7, HOXA9* and *HOXA11* were remarkably decreased after GSK-J4 treatment. ChIP-qPCR analyses verified that GSK-J4 suppressed the expression of these *HOX* genes through increasing the H3K27me3 levels in their TSS regions, further confirmed that the growth of AML cells can be blocked through enhancement of H3K27me3-mediated cancer-promoting gene suppression, such as *HOX* families.

## Conclusions

Our study showed that GSK-J4, a KDM6B inhibitor, blocked the growth of AML cells by inhibiting the expression of key regulators in DNA replication and cell cycle, as well as enhancing the global level of H3K27me3 which mediated the suppression of *HOX* genes. More importantly, administration of GSK-J4 reduced the tumor burden in a humanized murine model of AML in vivo. Taken together, these results provide significant evidence that targeting KDM6B with GSK-J4 has a therapeutic potential for the treatment of AML. This study sheds lights on the further investigation of targeting KDM6B with GSK-J4 or other small molecule inhibitors in the treatment of patients with AML.

## Electronic supplementary material

Below is the link to the electronic supplementary material.

Supplementary material 1-Online Resource 1 (DOCX 1946 KB)
