# Supplementary material for: Therapeutic potential of GSK-J4, a histone demethylase KDM6B/JMJD3 inhibitor, for acute myeloid leukemia
Source: J Cancer Res Clin Oncol. 2018 Mar 28;144(6):1065–77. doi: 10.1007/s00432-018-2631-7 (PMC5948279; doi:10.1007/s00432-018-2631-7)
Supplement: Supplementary file 1 — Supplementary material 1-Online Resource 1 (DOCX 1946 KB) [file 432_2018_2631_MOESM1_ESM.docx]

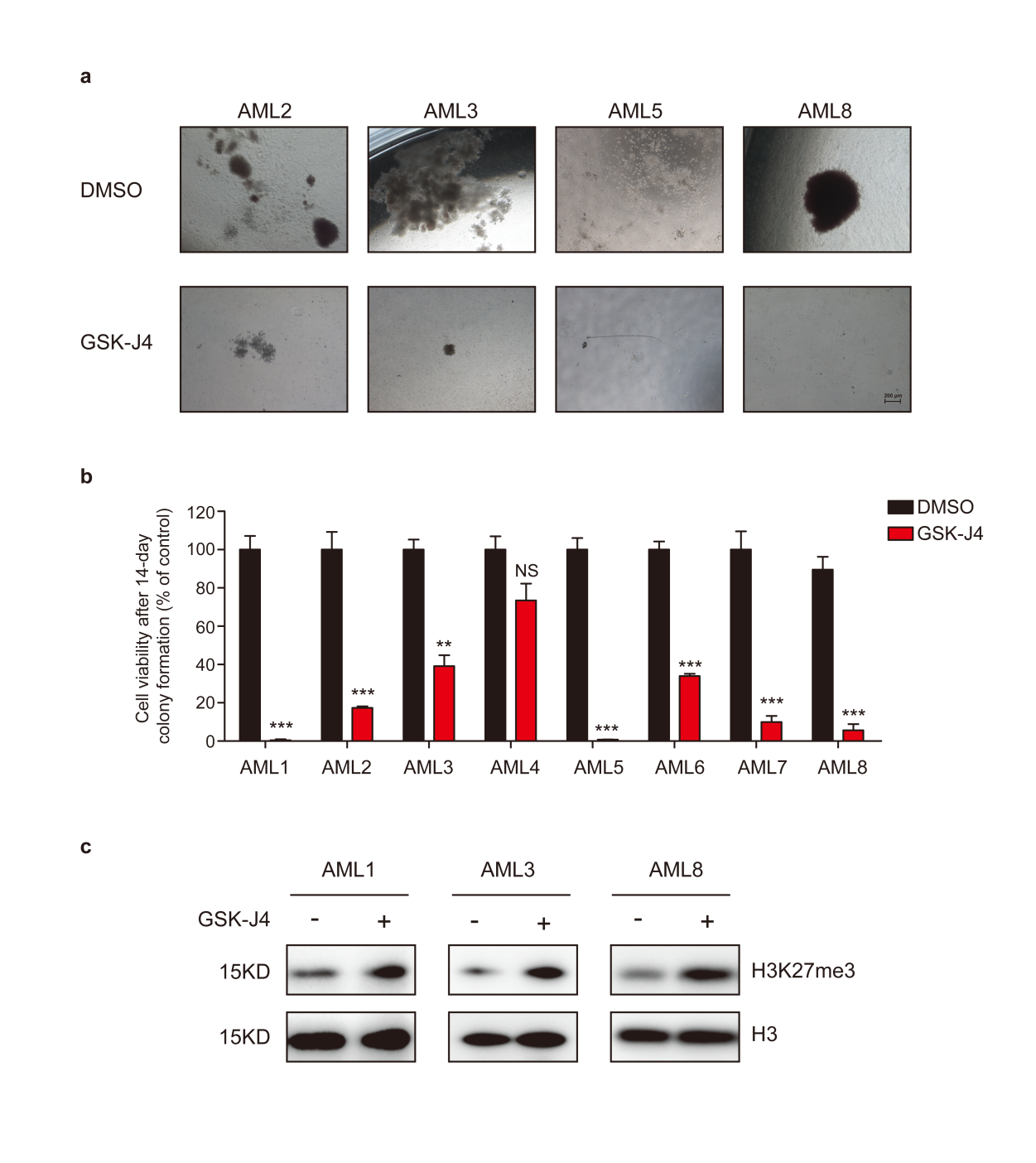
**Fig. S1 (Related to Fig. 2)** GSK-J4 treatment inhibits the proliferation and colony formation of primary AML cells. (**a**) The representative microscopy images of colony forming units (CFUs) in GSK-J4 or DMSO treated primary AML MNCs were shown. Scale bars represent 200 μm. (**b**) Total cell numbers of primary AML colonies with or without GSK-J4 treatment after 14-day methylcellulose culture were counted and normalized with the control group. Data represents mean ± SEM, ***P*<0.01, ****P*<0.001, NS: no significance, two-tailed Student *t*-test. (**c**) Western blot showed that GSK-J4 treatment (5.5 μM, 24 hours) led to increased H3K27me3 levels in primary AML MNCs.


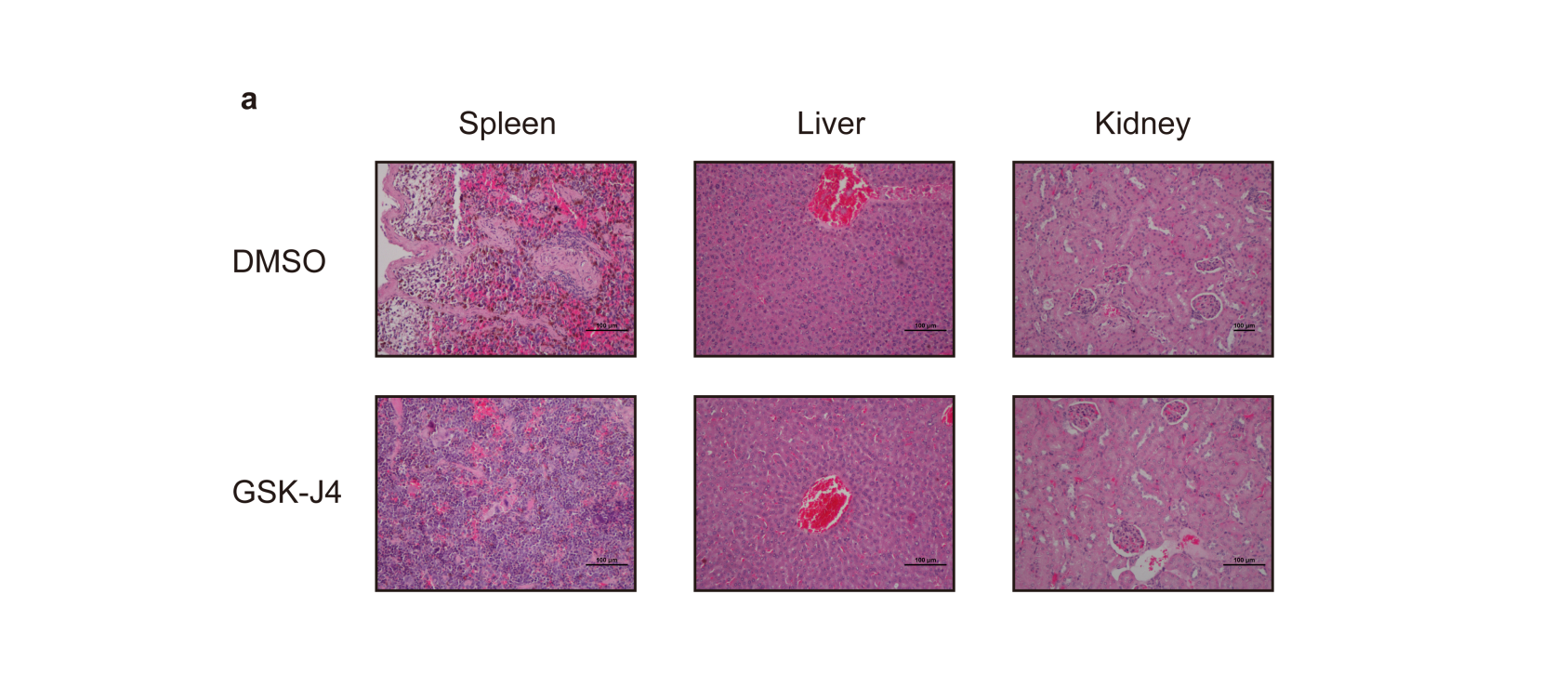


**Fig. S2 (Related to Fig. 4)** GSK-J4 treatment does not show apparent toxicity for NCG mice transplanted with Kasumi-1 cells. (**a**) Representative images of histological spleens, livers and kidneys sections by H&E staining from mice treated with DMSO or GSK-J4 were shown. Scale bars represent 100 μm.


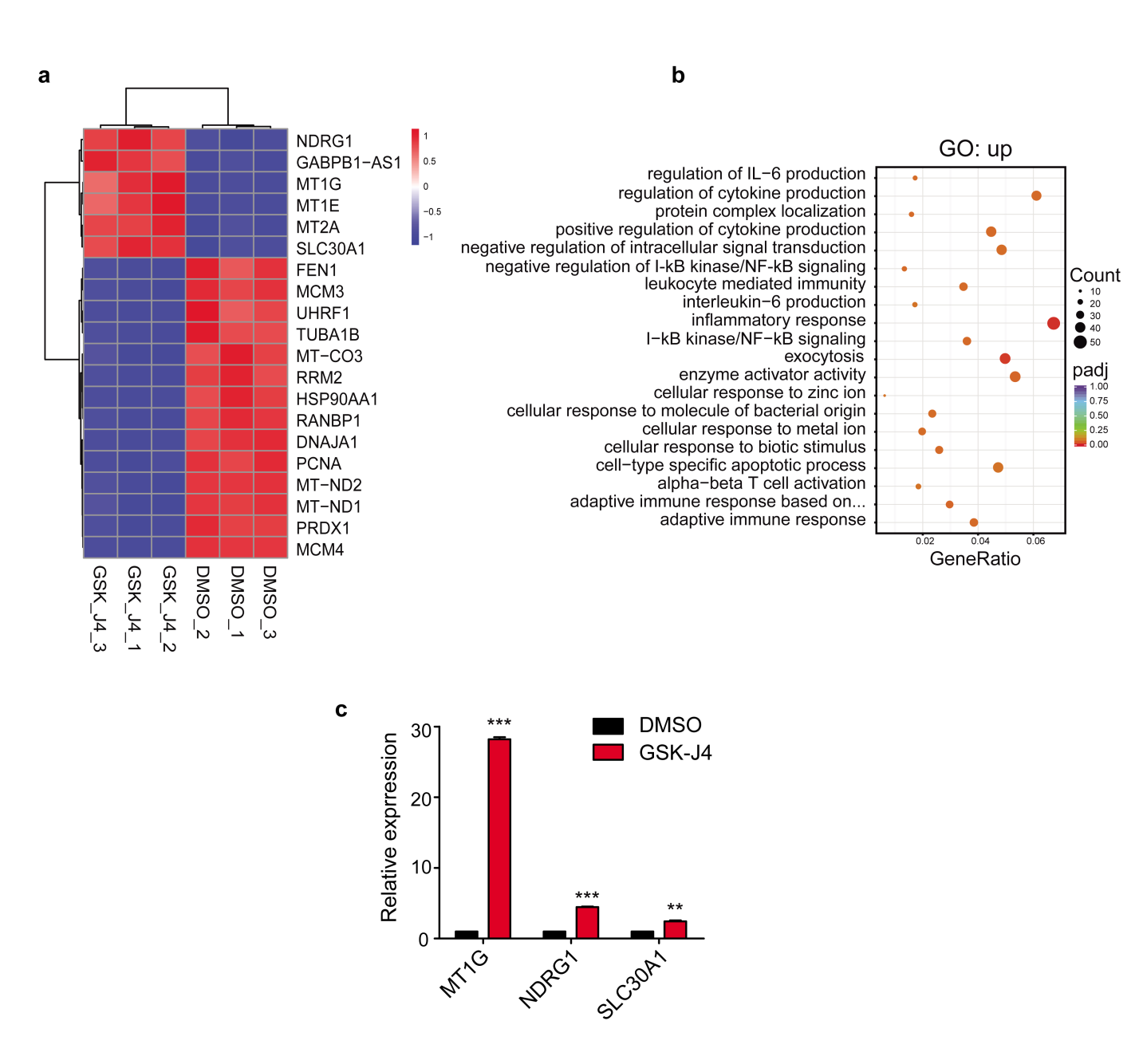
**Fig. S3 (Related to Fig. 5)** GSK-J4 treatment increases the expression of specific genes of Kasumi-1 cells. (**a**) Heatmap of the top-20 significantly differentially expressed genes between DMSO and GSK-J4 treated Kasumi-1 cells (n=3 per group, adjusted *P*<0.05, fold-change >1.6). (**b**) Dot plots showed the top-20 enriched GO terms for up-regulated genes. The adjusted *P-*value of “inflammatory response” and “exocytosis” are less than 0.05. The full description of the term “adaptive immune response based on…” is “adaptive immune response based on somatic recombination of immune receptors built from immunoglobulin superfamily domains”. (**c**) qPCR results showed the expression levels of key up-regulated genes in Kasumi-1 cells treated with GSK-J4. Gene expression was normalized to *GAPDH*. ***P*<0.01, ****P*<0.001, two-tailed Student *t*-test.

| **Sample** | **FAB** | **Disease Stage** | **Cytogenetics** | **Sex** | **Age(y)** | **PB/BM** | **Blasts(%)** |
| --- | --- | --- | --- | --- | --- | --- | --- |
| AML1 | M2a | Initial | Normal | Female | 7 | BM | 92.9 |
| AML2 | M2 | Initial | Normal | Male | 62 | BM | 61.8 |
| AML3 | M5a | Initial | del(11)(q23),?i(17)(q10) | Male | 25 | BM | 96 |
| AML4 | M5b | Initial | Normal | Male | 44 | BM | 93.9 |
| AML5 | M5 | Initial | 47,XY,+13 | Male | 70 | BM | 86.2 |
| AML6 | M0/M1 | Initial | Normal | Male | 22 | BM | 82.6 |
| AML7 | M2 | Initial | Normal | Male | 67 | BM | 57.3 |
| AML8 | M2b | Initial | NA | Male | 59 | BM | 33.7 |

**Table S1** Patient clinical characteristics

BM (bone marrow); NA (not available)

**Table S2** RT-qPCR Primers

|  | **Primer** | **Sequence (5’ to 3’)** |
| --- | --- | --- |
|  | FEN1-F | ATGACATCAAGAGCTACTTTGGC |
|  | FEN1-R | GGCGAACAGCAATCAGGAACT |
|  | HSP90AA1-F | AGGAGGTTGAGACGTTCGC |
|  | HSP90AA1-R | AGAGTTCGATCTTGTTTGTTCGG |
|  | MCM3-F | TCAGAGAGATTACCTGGACTTCC |
|  | MCM3-R | TCAGCCGGTATTGGTTGTCAC |
|  | MCM4-F | GACGTAGAGGCGAGGATTCC |
|  | MCM4-R | GCTGGGAGTGCCGTATGTC |
|  | PCNA-F | GCGTGAACCTCACCAGTATGT |
|  | PCNA-R | TCTTCGGCCCTTAGTGTAATGAT |
|  | MT1G-F | GGAACTCTAGTCTCGCCTCG |
|  | MT1G-R | GCATTTGCACTCTTTGCACT |
|  | NDRG1-F | CTCCTGCAAGAGTTTGATGTCC |
|  | NDRG1-R | TCATGCCGATGTCATGGTAGG |
|  | SLC30A1-F | CCCCGCAGACCCAGAAAAC |
|  | SLC30-R | ACGCATGTTAAGTTGTCCAGC |
|  | HOXA5-F | GCGCAAGCTGCACATAAGTC |
|  | HOXA5-R | GAACTCCTTCTCCAGCTCCA |
|  | HOXA11-F | ACACTGAGGACAAGGCCG |
|  | HOXA11-R | GAAGAAGAACTCCCGTTCCA |
|  | GAPDH-F | GAAGGTGAAGGTCGGAGTC |
|  | GAPDH-R | GAAGATGGTGATGGGATTTC |

**Table S3** ChIP-qPCR Primers

| **Primers** | **Position** | **Sequence(5’ to 3’)** |
| --- | --- | --- |
| HOXA5-F | chr7:27,143,701-27,143,721 | TGGGACATGTACTTGGTTCCC |
| HOXA5-R | chr7:27,143,826-27,143,845 | CCTCCACCCAACTCCCCTAT |
| HOXA11-F | chr7:27,185,009-27,185,028 | AGTATGTCATTGGGCGCGAA |
| HOXA11-R | chr7:27,185,108-27,185,127 | TGGTCCCTGCTCCTCTAACA |
